# Supplementary material for: Performance of Risk Scores in Predicting Infective Endocarditis in Patients with Staphylococcus aureus Bacteraemia in a Prospective Asian Cohort
Source: J Clin Med. 2024 May 16;13(10):2947. doi: 10.3390/jcm13102947 (PMC11122131; doi:10.3390/jcm13102947)
Supplement: Supplementary file 1 [file jcm-13-02947-s001.zip › jcm-2920608-supplementary.pdf]

Supplemental Table S1: Components of each clinical prediction tool of infective endocarditis in patients with *Staphylococcus aureus* bacteraemia

PREDICT Day 1 (cutoff ≥4)		PREDICT Day 5 (cut off ≥2)		VIRSTA (cutoff ≥3)	
Item	Points	Item	Points	Item	Points
Implantable cardioverter-defibrillator	2	Implantable cardioverter-defibrillator	2	Cerebral or peripheral emboli	5
Permanent pacemaker	3	Permanent pacemaker	3	Meningitis	5
Community acquisition	2	Community acquisition	2	Permanent intracardiac device or previous IE	4
Healthcare acquisition	1	Healthcare acquisition	1	Pre-existing native valve disease	3
		Positive blood culture after 72h	2	IV drug use	4
				Positive culture after 48h	3
				Community or healthcare-associated bacteraemia	2
				Severe sepsis or septic shock	1
				C-reactive protein >190mg/L	1
